# The effect of exercise training on the quality of sleep in national-level adolescent finswimmers

**DOI:** 10.1186/s40798-019-0207-y

**Published:** 2019-08-07

**Authors:** Vasileios Stavrou, George D. Vavougios, Fotini Bardaka, Eleni Karetsi, Zoe Daniil, Konstantinos I. Gourgoulianis

**Affiliations:** 1 Laboratory of Cardio-Pulmonary Testing, Department of Respiratory Medicine, Faculty of Medicine, University of Thessaly, Biopolis, Larissa, Greece; 2 Department of Neurology, Athens Naval Hospital, Deinokratous 70, Athens, Greece

**Keywords:** Finswimming, Sleep quality, Young athletes, Junior Championship

## Abstract

**Background:**

The purpose of the present study was to investigate whether the quality of sleep, in 91 national-level adolescent finswimmers, is affected by swimming style, swimming distance, and gender.

**Methods:**

Twenty-four hours before the opening of the National Championship, the participants recorded the training characteristics and answered the following two questionnaires: Pittsburgh Sleep Quality Index (PSQI) and Epworth Sleepiness Score. Athletes were allocated to groups by gender, swimming style (monofin vs. bifin) and swimming distance (≤ 200 m vs. > 200 m). The dependencies between qualitative variables were estimated by chi-square test or Cramer’s *V* test with modification by Fisher’s exact test with cell frequencies less than 5. Binary logistic regression was used in the multi-factor model.

**Results:**

There was a difference in the variables of PSQI “usual getting up time” and “have pain during sleep” between the two swimming distance groups (≤ 200 m vs. > 200 m). By using a multi-factor model (*χ*^2^ = 13.541, *p* = 0.035), the variables of PSQI “usual getting up time” and “have pain during sleep” remained independent predictors of the swimming distance (*p* = 0.019, OR 1.75, 95% CI 1.09–2.81).

**Conclusion:**

The athletes swimming distances > 200 m experience more episodes of pain during sleep and get up earlier than athletes swimming shorter distances.

## Key Points

- Sleep disturbances reflect symptoms of increasing training load such as preparation periods.
- The parameters of the PSQI questionnaire “early getting up” and “have pain” probably are a combination of leg fatigue due to heavy equipment.
- Athletes swimming middle-to-long distances experience more episodes of pain during sleep.

## Background

Finswimming is a speed competition sport practiced at the surface or underwater with monofin (MF) or bifins (BF) of variable rigidity. In MF, finswimmer’s lower limbs are used for propulsion purposes as well as the body’s vertical displacement while BF is crawl swimming on the breast with snorkel for breathing all the time at all the distances using fins [1]. In high-level athletes, sleep quality is affected by the kind of sport (individual or team), the type of exercise (aerobic, anaerobic, resistance, etc.), the training frequency [2], psychobiological mechanisms, and chronotype [3]. The psychobiological functions show maximum peaks at different times of the day, which could have either positive or negative effects on sports performance. Chronotype influences ratings of perceived exertion and improves athletic performances, as measured by race times, in the morning. According to Vitale et al. [3], athletes have better swimming performance in the morning session and this is related to “time since awakening,” which is an endogenous factor that clearly interacts with the external circumstances. These results show that what really matters for an athlete is chronotype and how many hours after entrained wake-up the competition or performance evaluation takes place [3].

The purpose of the study was to investigate whether the quality of sleep, in high national-level adolescent finswimmers, is affected by swimming style, swimming distance, or gender.

## Methods

### Participants

During the National Finswimming Junior Greek Championship, 91 high-level finswimmers, from 10 sports clubs, participated in the study (Table 1). Inclusion criteria were athletes aged between 14 and 18, training age ≥ 2 years and not a recent injury (≤ 30 days). The study was conducted according to the Helsinki declaration for use in human subjects (No 58076/14-11-2018, Scientific Council of University Hospital of Larissa, Greece). All athletes consented to participate in the study, but, since they are minors, the consent was given by coaches, team chiefs, and parents (in addition to the written consent obtained from the caregivers).

**Table 1:** Results between groups (mean ± SD)

	Athletes	Gender			Swimming style			Swimming distance		
	Total *n* = 91	M *n* = 41	F *n* = 50	*P* value	BF *n* = 38	MF *n* = 53	*P* value	≤ 200 m *n* = 52	> 200 m *n* = 39	*P* value
Age, years	15.8 ± 1.3	16.1 ± 1.4	15.5 ± 1.2	0.027	16.0 ± 1.4	15.6 ± 1.2	NS	15.8 ± 1.1	15.8 ± 1.5	NS
Training age, years	3.8 ± 2.0	4.0 ± 1.7	3.7 ± 2.2	NS	2.9 ± 1.4	4.5 ± 2.1	< 0.001	3.7 ± 1.8	4.1 ± 2.1	NS
Training/day/min^−1^	113.2 ± 22.1	114.1 ± 24.2	112.4 ± 20.5	NS	118.4 ± 18.4	109.5 ± 23.8	NS	114.2 ± 19.7	111.8 ± 25.1	NS
Training/week, frq	5.9 ± 1.4	5.5 ± 1.4	6.1 ± 1.5	NS	5.9 ± 1.6	5.8 ± 1.2	NS	5.6 ± 1.3	6.2 ± 1.5	NS
Gym/day/min^−1^	76.1 ± 21.7	78.6 ± 21.9	74.0 ± 21.4	NS	73.7 ± 21.2	77.8 ± 22.0	NS	79.2 ± 20.6	71.9 ± 22.6	NS
Gym/week, frq	3.0 ± 0.9	2.9 ± 0.7	3.1 ± 1.1	NS	2.8 ± 0.5	3.2 ± 1.1	0.024	2.9 ± 0.6	3.2 ± 1.2	NS
Diet, % of yes	45%	24%	18%	NS	20%	25%	NS	22%	23%	NS
Sport dietary supplements, % of yes	77%	30%	35%	0.006	35%	43%	NS	43%	32%	NS
ESS, score	3.0 ± 1.1	3.0 ± 1.0	3.1 ± 1.2	NS	2.9 ± 1.1	3.2 ± 1.1	NS	3.2 ± 1.0	2.9 ± 1.2	NS

*Abbreviations*: *BF* bifins, *ESS* Epworth Sleepiness Score, *F* female, *frq* frequency, *M* male, *MF* monofin

### Data Collection

Twenty-four hours before the opening of the National Finswimming Junior Greek Championship, the participants recorded the training characteristics (training frequency and training hours in pool and gym respectively), the diet guidance from nutritionists (yes vs. no), and the use of sports dietary supplements (yes vs. no) and answered the following two questionnaires: Pittsburgh Sleep Quality Index (PSQI) [4] and Epworth Sleepiness Score [5]. Athletes were allocated to groups by gender [male (M) vs. female (F)], swimming style [monofin (MF) vs. bifins (BF)], and swimming distance [≤ 200 m (sprints) vs. > 200 m (middle-to-long distances)].

### Statistical Analysis

Kolmogorov-Smirnov test was used for the normality of the distribution. The independent samples *t* test was used for statistical comparison between groups (gender, female vs. male; finswimming style, BF vs. MF; swimming distance, ≤ 200 m vs. > 200 m). The dependencies between qualitative variables were calculated by chi-square test or Cramer’s *V* test with modification by Fisher’s exact test with cell frequencies less than 5. Binary logistic regression was used for the multi-factor model. For each test, the level of significance was set to *p* < 0.05 and the data are presented as mean value and standard deviation (mean ± SD). The SPSS 15 statistical package (SPSS Inc., Chicago, Illinois, USA) was used for the statistical analyses.

## Results

The results between groups for the independent samples *t* test are presented in Table 1. The results of PSQI (Table 2) showed a difference in variables “usual getting up time” and “have pain during sleep” in swimming distance (≤ 200 m vs. > 200 m) between groups. Results did not show any difference between swimming style (MF vs. BF, *p* > 0.05), gender (M vs. F, *p* > 0.05), and age (*p* > 0.05). By using a multi-factor model (*χ*^2^ = 13.541, *p* = 0.035), the variables of PSQI “usual getting up time” and “have pain during sleep” remained independent predictors of the swimming distance (*p* = 0.019, OR 1.75, 95% CI 1.09–2.81).

**Table 2:** PSQI results between groups (mean ± SD)

		Gender			Swimming style			Swimming distance		
		M *n* = 41	F *n* = 50	*P* value	BF *n* = 38	MF *n* = 53	*P* value	≤ 200 m *n* = 52	> 200 m *n* = 39	*P* value
1	When have you usually gone to bed?	10.3 ± 0.9	9.4 ± 1.0	NS	10.5 ± 1.1	10.1 ± 1.5	NS	10.2 ± 1.4	11.0 ± 2.0	NS
2	How long has it taken you to fall asleep each night?	20.2 ± 14.0	20.2 ± 18.7	NS	19.2 ± 11.5	20.9 ± 19.6	NS	22.3 ± 19.8	17.8 ± 12.2	NS
3	What time have you usually gotten up in the morning?	9.2 ± 1.4	9.6 ± 1.8	NS	9.0 ± 1.6	9.7 ± 1.6	NS	9.1 ± 1.6	9.5 ± 1.7	NS
4	How many hours of actual sleep did you get at night?	8.3 ± 1.4	8.0 ± 1.8	NS	7.8 ± 1.8	8.4 ± 1.5	0.022	7.9 ± 1.7	8.2 ± 1.5	0.022
5	Cannot get to sleep within 30 min	1.0 ± 1.2	1.1 ± 1.1	NS	1.2 ± 1.2	1.0 ± 1.1	NS	1.1 ± 1.2	1.0 ± 1.0	NS
6	Wake up in the middle of the night or early morning	1.1 ± 1.0	1.3 ± 1.1	0.035	1.3 ± 1.1	1.2 ± 1.0	NS	1.4 ± 1.0	1.0 ± 1.2	NS
7	Have to get up to use the bathroom	0.9 ± 1.1	0.8 ± 1.1	NS	0.6 ± 0.9	1.1 ± 1.1	NS	1.0 ± 1.1	0.6 ± 0.9	NS
8	Cannot breathe comfortably	0.3 ± 0.6	0.4 ± 0.9	0.030	0.5 ± 0.9	0.3 ± 0.6	NS	0.5 ± 0.8	0.2 ± 0.6	NS
9	Cough or snore loudly	0.3 ± 0.7	0.2 ± 0.5	NS	0.2 ± 0.6	0.3 ± 0.6	NS	0.3 ± 0.5	0.2 ± 0.7	NS
10	Feel too cold	0.3 ± 0.6	0.7 ± 0.8	NS	0.5 ± 0.8	0.5 ± 0.7	NS	0.5 ± 0.8	0.5 ± 0.7	NS
11	Feel too hot	1.7 ± 1.0	1.5 ± 1.1	NS	1.5 ± 1.2	1.7 ± 1.0	NS	1.8 ± 1.0	1.3 ± 1.1	NS
12	Have bad dreams	0.6 ± 0.8	0.9 ± 0.8	NS	0.8 ± 0.8	0.8 ± 0.9	NS	0.7 ± 0.8	0.8 ± 0.8	NS
13	Have pain	0.5 ± 0.8	0.9 ± 1.0	NS	0.7 ± 1.0	0.7 ± 1.0	NS	0.5 ± 0.8	1.0 ± 1.1	0.010
14	During the past month, how often have you taken medicine to help you sleep?	0.2 ± 0.6	0.3 ± 0.8	NS	0.2 ± 0.6	0.2 ± 0.7	NS	0.1 ± 0.5	0.2 ± 0.7	NS
15	During the past month, how often have you had trouble staying awake while driving, eating meals, or engaging in social activity?	0.4 ± 0.7	0.7 ± 0.9	NS	0.5 ± 0.8	0.6 ± 0.9	NS	0.6 ± 0.8	0.4 ± 0.7	NS
16	During the past month, how much of a problem has it been for you to keep up enthusiasm to get things done?	0.9 ± 1.0	1.1 ± 0.8	NS	1.0 ± 0.8	1.0 ± 0.9	NS	1.0 ± 0.9	1.1 ± 0.9	NS
17	During the past month, how would you rate your sleep quality overall?	0.9 ± 0.9	1.2 ± 0.7	NS	1.1 ± 0.7	1.0 ± 0.8	NS	1.0 ± 0.7	1.2 ± 0.8	NS

Questions 5–13 [scale: not during the past month (0), less than once a week (1), once or twice a week (2), three or more times a week (3)]; questions 14–17 [scale: very good (0), fairly good (1), fairly bad (2), very bad (3)]

*Abbreviations*: *BF* bifins, *F* female, *M* male, *MF* monofin

## Discussion

The data from the present study reveal that athletes swimming distances more than 200 m show more episodes of pain during sleep compared to athletes racing shorter distances, which is likely to be responsible for “usual getting up time” earlier in the morning. Sleep is a key component in an athlete’s recovery process while sleep disturbances are believed to reflect symptoms of overreaching or overtraining in periods of increasing training load such as pre-competition periods [6]. The influences of the chronotype on sleep quality are related to high-intensity interval training and the time of game, and these should be taken into account when scheduling training sessions in order to promote faster recovery processes [7]. Sleeping patterns may vary throughout the season, depending on the competition period but are related to muscle and bone injuries [8]. According to Carskadon [9], sleep loss in adolescents is influenced by screen time, technology use, and social engagement in the evening and is a multi-factorial situation that is associated with a convergence of biological, psychological, and socio-cultural influences. Chronic or acute sleep loss is directly correlated with athletic injuries and/or “fatigue-related injuries,” and this reduced amount of sleep is a direct, independent risk factor for injuries during exercise, while sleep deprivation increases the risk of over-strain injuries and impairs the functional recovery of muscles following injury [10].

In the present study, the adolescent athletes filled in the PSQI questionnaire 24 h before the opening of the National Finswimming Junior Greek Championship. The PSQI scores are poorest during the competition phase, whereas athletes appear to be sleepier during the day and/or have a greater sleep requirement due to training loads and intensity [11]. Our results showed that athletes were classified as good sleepers at the total PSQI score (4.7 ± 3.0) and swimming distances (> 200 m 4.8 ± 2.8 vs. ≤ 200 m 4.6 ± 3.3), compared to previous studies where athletes were classified as poor sleepers when having score > 5.5 in PSQI [4, 12]. According to Swinbourne et al. [11], highly trained team-sport athletes report longer sleep times during the off-season compared to athletes during competition phases. The performances in competition are related to sleep quality providing indirect information about the intensity during the specific and race preparation period and about the physical and emotional exhaustion of the athletes. Moreover, during periods where training loads are high, some athletes report difficulties falling asleep, restlessness during sleep, and heavy legs during sleep, which are physiological symptoms of overtraining syndrome [6].

The parameters “early getting up” and “have pain” probably are a combination of leg fatigue, due to heavy equipment (MF: fin with approximate weight of 3.5 kg^−1^; and BF: two fins with approximate weight of 1.1 kg^−1^) [13] and disturbed sleep due to thoughts about competition and nervousness [14]. These athletes (middle-to-long distance) may be more concerned about the strategies/tactics of the competition, in combination with pre-competition anxiety [15] as is usually the case between sprinters and long-distance athletes [16].

## Limitations

Nevertheless, in our study there were some limitations. The participants aged between 14 and 18 and possibly the different stage of puberty could affect their behavioral development and the different sleep patterns. Furthermore, chronotype, a factor that influences athletes’ sleep, was not evaluated [17].

## Conclusion

The present study shows that athletes swimming middle-to-long distances (> 200 m) experience more episodes of pain during sleep and this is responsible for “usual getting up time” compared to athletes swimming shorter distances (≤ 200 m).

## Data Availability

Please contact author for data requests.

## Abbreviations

BF: Bifins
ESS: Epworth Sleepiness Score
F: Female
Frq: Frequency
M: Male
MF: Monofin
PSQI: Pittsburgh Sleep Quality Index

## References

[1] Stavrou V, Vavougios G, Karetsi E, Adam G, Daniil Z, Gourgoulianis KI (2018). Evaluation of respiratory parameters in finswimmers regarding gender, swimming style and distance. Respir Physiol Neurobiol..

[2] Nedelec M, Aloulou A, Duforez F, Meyer T, Dupont G (2018). The variability of sleep among elite athletes. Sports Med Open..

[3] Vitale JA, Weydahl A (2017). Chronotype, physical activity, and sport performance: a systematic review. Sports Med..

[4] Driller MW, Mah CD, Halson SL (2018). Development of the athlete sleep behavior questionnaire: A tool for identifying maladaptive sleep practices in elite athletes. Sleep Sci..

[5] Johns MW (1991). A new method for measuring daytime sleepiness: the Epworth sleepiness scale. Sleep..

[6] Lastella M, Vincent GE, Duffield R, Roach GD, Halson SL, Heales LJ, Sargent C (2018). Can sleep be used as an indicator of overreaching and overtraining in athletes?. Front Physiol..

[7] Vitale JA, Bonato M, Galasso L, La Torre A, Merati G, Montaruli A, Roveda E, Carandente F (2017). Sleep quality and high intensity interval training at two different times of : a crossover study on the influence of the chronotype in male collegiate soccer players. Chronobiol Int..

[8] Vitale JA, Banfi G, La Torre A, Bonato M (2018). Effect of a habitual late-evening physical task on sleep quality in neither-type soccer players. Front Physiol..

[9] Carskadon MA (2011). Sleep in adolescents: the perfect storm. Pediatr Clin North Am..

[10] Chennaoui M (2015). ArnalPJ, Sauvet F, Leger, D. Sleep and exercise: a reciprocal issue?. Sleep Med Rev..

[11] Swinbourne R, Gill N, Vaile J, Smart D (2016). Prevalence of poor sleep quality, sleepiness and obstructive sleep apnoea risk factors in athletes. Eur J Sport Sci..

[12] Mah CD (2018). KezirianEJ, Marcello BM, Dement WC. Poor sleep quality and insufficient sleep of a collegiate student-athlete population. Sleep Health..

[13] Stavrou V, Tsarouhas K, Karetsi E, Michos P, Daniil Z, Gourgoulianis K (2018). Adolescent finswimmers: early myocardial adaptations in different swimming styles. Sports (Basel)..

[14] Hatzigeorgiadis A, Chroni S (2007). Pre-competition anxiety and in-competition coping in experienced male swimmers. Int J Sports Sci Coach..

[15] Erlacher D, Ehrlenspiel F (2011). AdegbesanOA, El-Din HG. Sleep habits in German athletes before important competitions or games. J Sports Sci..

[16] Maruo Y, Murphy IT, Masaki H (2018). Long-distance runners and sprinters show different performance monitoring - an event-related potential study. Front Psychol..

[17] Vitale JA, Banfi G, Sias M, La Torre A (2019). Athletes’ rest-activity circadian rhythm differs in accordance with the sport discipline. Chronobiol Int..

